# Remote Cerebellar Hemorrhage Associated With Intra-Operative Cerebrospinal Fluid Leak: A Report of Two Rare Case Presentations and Review of the Literature

**DOI:** 10.7759/cureus.12082

**Published:** 2020-12-14

**Authors:** Long Di, Grace Wei, Daniel G Eichberg, Ricardo J Komotar, Michael Ivan

**Affiliations:** 1 Neurological Surgery, University of Miami Miller School of Medicine, Miami, USA; 2 Neurological Surgery, Sylvester Comprehensive Cancer Center, Miami, USA

**Keywords:** remote cerebellar hemorrhage, rch, cerebrospinal fluid, suboccipital, burr hole, subdural hygroma

## Abstract

Remote cerebellar hemorrhage (RCH) is a rare complication following cranial or spinal neurosurgical procedures. Traditionally, RCH has been associated with frontal or frontotemporal craniotomy with supine patient positioning. Though the exact etiology is unknown, theories have described patient positioning and excessive cerebrospinal fluid (CSF) drainage intra-operatively as contributing factors to cerebellar displacement (cerebellar sag), obstruction of venous flow, and pathogenesis of RCH.

We report two cases of RCH following a prone, suboccipital craniotomy-C1 laminectomy and a temporal burr hole evacuation of a subdural hygroma. In each case, a large volume of CSF was rapidly evacuated intra-operatively. To the best of our knowledge, both instances represent relatively rare settings for RCH. Additionally, we conducted a comprehensive literature review of PubMed, EMBASE, and Web of Science for all cases of RCH in which peri-operative CSF leakage was explicitly detailed.

Although RCH is thought to be a rare complication of frontotemporal and frontal craniotomies, this case report signifies that RCH may occur in the setting of sub-occipital craniotomy or even after minimally invasive burr hole procedures. For these procedures, careful symptomatic monitoring and follow-up imaging remain essential in diagnosis. Controlled CSF drainage may be useful in mediating dramatic alterations in intracranial pressure (ICP) and cerebellar sag contributing to RCH.

## Introduction

Remote cerebellar hemorrhage (RCH) is a rare intracerebral hemorrhage isolated from surgical sites following cranial or spinal procedures that may result in significant peri-operative neurologic morbidity and mortality [1]. Diagnosis involves detection of neurologic symptoms, commonly impaired consciousness, followed by identification of a “zebra sign” on axial head CT [1]. The pathogenesis and pathophysiology of RCH remain puzzling and several mechanisms explaining its etiology have been described, including cerebrospinal fluid (CSF) egress causing “cerebellar sag” and kinking of posterior venous sinuses [2]. Interestingly, RCH seems to occur most frequently following frontal or frontotemporal craniotomies contralateral to the side of RCH with supine patient positioning [3]. We report two rare presentations of RCH following sub-occipital craniotomy for a foramen magnum meningioma and burr hole evacuation of a temporal cystic hygroma. To our knowledge, this is one of the first reports of RCH following prone, sub-occipital craniotomy, and minimally invasive burr hole procedure for hygroma evacuation. In both cases, rapid and extensive CSF evacuation occurred. Additionally, we present a literature review of all reported RCH cases following craniotomy or spine procedure with explicitly stated peri-operative CSF leakage.

## Case presentation

Case 1

**Figure 1:**
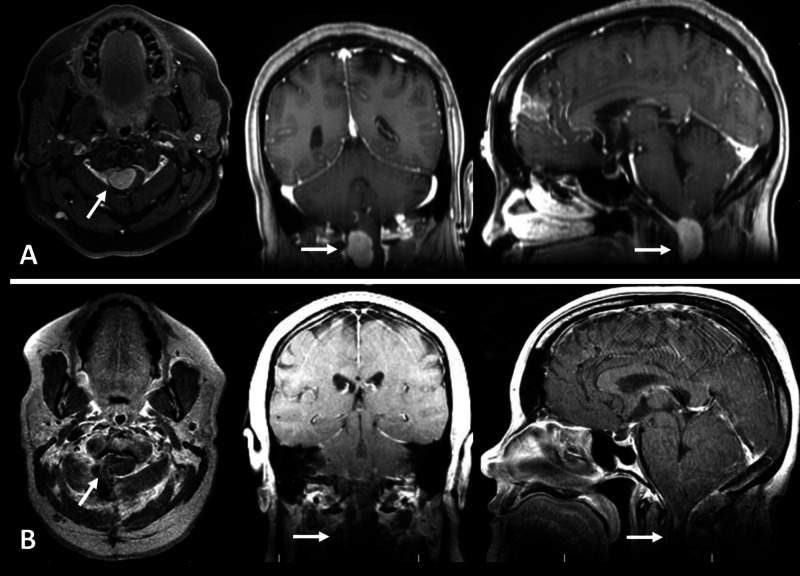
Pre-operative and post-operative T1 post-gadolinium contrast (T1C+) MRIs of a foramen magnum meningioma resected via sub-occipital craniotomy. (A) Pre-operative axial, coronal, and sagittal T1C+ images. (B) Post-operative images following gross total resection of meningioma.

A 55-year-old female presented to our clinic with several months history of right-sided facial numbness and left upper extremity numbness and tingling that progressed to the right upper extremity. She was referred by an outside neurologist following MRI that demonstrated a large, right-sided foramen magnum lesion compressing the spinal cord and brainstem. Imaging revealed a homogenously enhancing, extramedullary, intradural mass extending from the level of the foramen magnum to the mid-C2 vertebral body with an associated dural tail (Figures 1A, 1B).

**Figure 2:**
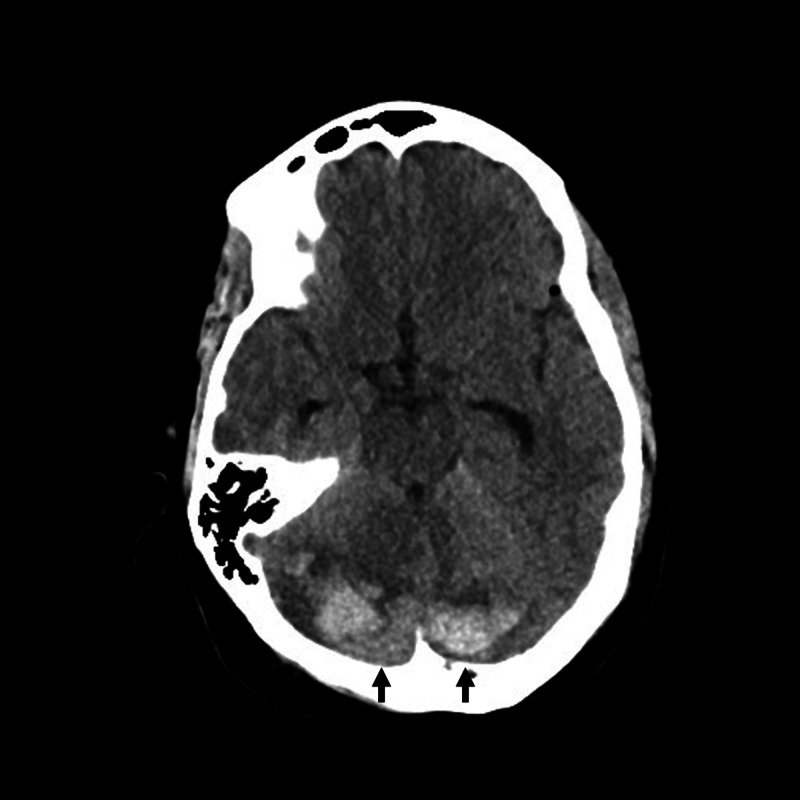
Axial CT indicating post-operative day one bilateral RCH in the superior cerebellum. RCH: remote cerebellar hemorrhage

Significant mass effect, compression, and displacement of the corticomedullary junction and upper cervical cord were identified. A working diagnosis of foramen magnum meningioma was made and the patient was scheduled for resection of the lesion. A sub-occipital craniotomy and C1-C2 laminectomy were performed with the patient in the prone position. During the exposure, standard arachnoid dissection facilitated CSF egress permitting a maximal safe resection of the tumor with minimal cerebellar retraction. On a post-operative day (POD) one, the patient developed nausea, vomiting, and positional headache exacerbated while lying flat. CT showed an interval increase in ventricular size with new cerebellar hyperdensity concerning hemorrhage and effacement of the fourth ventricle (Figure 2).

A diagnosis of acute obstructive hydrocephalus was made, and an emergency right frontal external ventricular drain (EVD) was placed. The RCH stabilized, hydrocephalus resolved, and EVD was removed on POD 10; the patient was discharged on POD 14. At one-month follow-up, the patient displayed a complete resolution of symptoms following physical rehabilitation; strength was 5/5 in bilateral upper and lower extremities with a Karnofsky Performance Status (KPS) of 90.

Case 2

**Figure 3:**
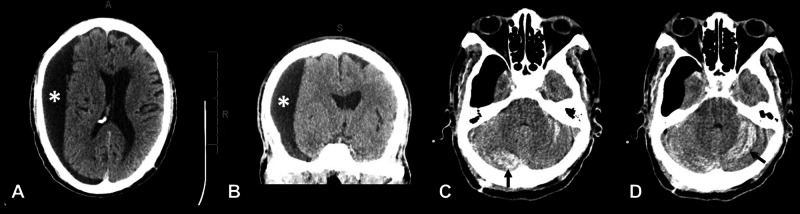
Case of bilateral RCH following burr hole evacuation of a right cystic hygroma. (A & B) Large right holohemispheric subdural hygroma measuring 2.8 cm in widest diameter with right hemispheric sulcal effacement and 10 mm of leftward midline shift. (C & D) s/p right-sided burr holes and placement of a subdural drain noted on postop CT were new bilateral cerebellar hemorrhages. *Right subdural cystic hygroma. RCH: remote cerebellar hemorrhage

A 79-year-old man with a history of hypertension, diabetes, hyperlipidemia, and aspirin use presented to our clinic with a three-day history of worsening generalized weakness and headache. The patient denied any symptoms other than weakness and slight tremor. Prior head CT displayed a large right-sided, hollow hemispheric hygroma with approximately 1 cm midline shift. The patient denied prior head trauma and no other focal neurologic deficits were observed. After five days of aspirin cessation, the patient underwent hygroma evacuation. Two burr holes were made and, following the dural opening, high-pressure CSF was immediately evacuated (Figures 3A, 3B).

A Jackson-Pratt (JP) drain was placed over the posterior burr hole followed by closure. The patient awoke on POD one able to follow commands but over subsequent hours, decompensated and had a tonic-clonic seizure. Head CT revealed a new cerebellar hemorrhage and the patient was taken for emergent suboccipital decompression (Figures 3C, 3D). On POD nine, the patient was able to open his eyes, weakly withdrew bilateral upper extremities to pain, and moved bilateral lower extremities spontaneously. Unfortunately, the patient’s condition continued to deteriorate. On POD 27, the patient had not recovered neurologically, remaining intubated and dependent on tube feeding; the decision to pursue palliative care was made and the patient was discharged to a nursing home.

## Discussion

A comprehensive literature review was performed in PubMed, EMBASE, and Web of Science databases to identify RCH cases with perioperative CSF leakage. Keywords included: remote, cerebellar, and hemorrhage. All abstracts were reviewed, and relevant articles were collected using the following inclusion and exclusion criteria. All articles in the English language presenting cases of RCH with perioperative CSF leakage were included. Review articles, letters and commentary, and cases without specific mention of perioperative CSF loss were excluded.

Including our own, twelve cases of RCH following craniotomy with peri-operative CSF leakage were identified [4-10]. The average patient age was 58.25 years. Nine patients were male and three were female. The majority of cases were pterional craniotomies (4) and temporal (3). In cases providing positioning detail, all patients were placed supine. Post-operative clinical findings included altered mental status (7), nausea, dysarthria, seizure, hemineglect, hemiplegia, headache, and ataxia; one case was identified incidentally on follow-up imaging. On imaging, bilateral and unilateral RCH were identified in eight and four patients, respectively. Typical “zebra” hemorrhage patterns were seen in most cases (Figure 3D). In addition, 58.3% of patients exhibited complete symptom resolution following decompression (7) with a motor deficit (2) and death (2) in 16.7% of patients, respectively. These cases are summarized in Table 1.

**Table 1:** Characteristics of Cranial Cases RCH: remote cerebellar hemorrhage; M: male; F: female; L: left; R: right; Mi: midline

#	Reference	Age	Sex	Side	Location of Craniotomy	Position of Patient	Clinical Findings	Post-Operative Hemorrhage	Follow-Up (months)	Outcome	Proposed Mechanism
1	Current study (Case 1)	55	F	Mi	Suboccipital	Prone	Nausea	Bilateral RCH	1	Complete resolution	Excessive CSF loss
2	Current study (Case 2)	79	M	R	Temporal (burr hole)	Prone	Tonic clonic seizure	Bilateral RCH	0.3	Coma	Excessive CSF loss
3	Caldeira et al., 2017 [4]	70	M	-	Frontal	Supine	None	Unilateral RCH	0.5	Complete resolution	Excessive CSF loss
4	Koh et al., 2017 [6]	62	M	L	Pterional	-	Altered mental status	Bilateral RCH	0.5	Death	Excessive CSF loss
5	Li et al., 2013 [7]	51	M	L	Pterional	-	Altered mental status	Unilateral RCH	3	Complete resolution	Excessive CSF loss
6	Li et al., 2013 [7]	67	F	L	Pterional	-	Altered mental status	Bilateral RCH	3	Complete resolution	Excessive CSF loss
7	Ziyal et al., 2012 [10]	30	M	L	Temporal	-	Headache, ataxia	Unilateral RCH	0.25	Complete resolution	Excessive CSF loss
8	De Ribaupierre et al., 2004 [5]	8	M	L	Periinsular	Supine; head rotated right	Altered mental status	Bilateral RCH	25	Complete resolution	Excessive CSF loss
9	Maruyama et al., 2004 [8]	83	M	L	Frontotemporal	Supine; head rotated right	Dysarthria	Bilateral RCH	0.5	Complete resolution	Excessive CSF loss
10	Siu et al., 2003 [9]	54	M	L	Pterional	Head rotated right	Altered mental status, hemiplegia, hemineglect	Bilateral RCH	1	Upper limb and facial paresis, dysarthria	Excessive CSF loss
11	Siu and Chandran, 2003 [9]	64	M	R	Temporal	Head tilted left	Altered mental status	Bilateral RCH	0.3	Death	Excessive CSF loss
12	Siu and Chandran, 2003 [9]	76	F	R	Parietotemporal	Lateral	Altered mental status	Unilateral RCH	2	Upper limb weakness	Excessive CSF loss

Seven cases of RCH following spinal surgery with peri-operative CSF leak were identified [11-16]. The average patient age was 61.57 years. Three patients were male and four were female. All cases were lumbar surgeries (7). Patient body positioning on all available cases (4) was prone. Peri-operative CSF leaks following intradural lesions were observed in 85.7% of patients (6), with CSF leaks after extradural pathology and dural tear observed in only 14.3% of patients (1). Post-operative clinical findings included altered mental status (6), nausea, emesis, dysarthria, seizure, headache, and diplopia. On imaging, bilateral and unilateral RCH was identified in six and one patient(s), respectively. Long-term patient outcomes included complete symptom resolution in 57.1% of patients (4), with a motor deficit in 28.6% (2) and death in 14.3% of patients (1). Table 2 represents all reports of RCH following spinal surgery included in this review; data for both cranial and spinal cases are statistically summarized in Tables 3, 4, respectively.

**Table 2:** Characteristics of Spinal Cases RCH: remote cerebellar hemorrhage; M: male; F: female

#	Reference	Age	Sex	Location of Spine Surgery	Position of Patient	Presence of Dural Tear	Clinical Findings	Post-Operative Hemorrhage	Follow-Up (months)	Outcome	Proposed Mechanism
1	Khalatbari et al, 2012 [13]	53	M	Lumbar	Prone	No	Headache, emesis, altered mental status	Bilateral RCH	3	Complete Resolution	Excessive CSF loss
2	Khalatbari et al., 2012 [13]	75	M	Lumbar	Prone	Yes	Altered mental status	Bilateral RCH	0.5	Death	Excessive CSF loss
3	Lee et al., 2012 [15]	63	F	Lumbar	-	Yes	Generalized tonic-clonic seizures, altered mental status	Bilateral RCH	0.4	Complete Resolution	Excessive CSF loss
4	Gul et al., 2010 [12]	64	F	Lumbar	Prone	Yes	Diplopia, altered mental status	Unilateral RCH	0.7	Gait ataxia, left foot drop	Excessive CSF loss
5	Nam et al., 2009 [16]	61	M	Lumbar	-	Yes	Headache, nausea, altered mental status	Bilateral RCH	1.5	Mild cerebellar signs	Excessive CSF loss
6	Calisaneller et al., 2007 [11]	67	F	Lumbar	-	Yes	Headache, gait ataxia, unsteadiness	Bilateral RCH	-	Complete Resolution	Excessive CSF loss
7	Konya et al., 2006 [14]	48	F	Lumbar	Prone	Yes	Headache, dysarthria, emesis, altered mental status	Bilateral RCH	6	Complete Resolution	Excessive CSF loss

**Table 3:** Summary of Cranial Cases SD: standard deviation

Characteristic	Total (%)
Total Patients	12
Male	9 (75)
Female	2 (25)
Age (mean±SD)	58.25±21.37
Craniotomy Side	
Left	7 (58.3)
Right	3 (25)
Midline	1 (8.3)
Craniotomy Location	
Pterional	4 (33.3)
Temporal	3 (25)
Other	5 (41.7)
Presentation	
Altered Mental Status	7 (58.3)
Other	5 (41.7)
Imaging	
Bilateral	8 (66.7)
Unilateral	4 (33.3)
Outcome	
Complete Resolution	7 (58.3)
Motor Deficit	2 (16.7)
Death	2 (16.7)
Recovering	1 (8.3)

**Table 4:** Summary of Spinal Cases SD: standard deviation

Characteristic	Total (%)
Total Patients	7
Male	3 (42.9)
Female	4 (57.1)
Age (mean±SD)	61.57±8.9
Spinal Surgery Level	
Lumbar	7 (100)
Presence of Dural Tear	
Yes	6 (85.7)
No	1 (14.3)
Presentation	
Altered Mental Status	6 (85.7)
Other	1 (14.3)
Imaging	
Bilateral	6 (85.7)
Unilateral	1 (14.3)
Outcome	
Complete Resolution	4 (57.1)
Motor Deficit	2 (28.6)
Death	1 (14.3)

Remote cerebellar hemorrhage is a rare complication following cranial and spinal surgeries with an estimated incidence of 0.08-0.6% [1]. RCH likely occurs due to rapid perioperative excess CSF loss leading to post-operative hemorrhagic venous infarction. Substantial CSF loss may result in “cerebellar sag,” with cerebellar shift downward; subsequent stretching of cerebellar short bridging veins may cause transient occlusion, leading to venous infarction and hemorrhage [3]. It remains unclear if RCH is precipitated peri-operatively or post-operatively, though most cases are identified immediately following surgery [3]. Indeed, both of our cases presented with neurologic symptoms and axial CT indicating RCH on a post-operative day one. Thus, post-operative monitoring and follow-up imaging may be essential in diagnosis and treatment with suboccipital decompression - even after procedures not traditionally associated with RCH.

Several symptoms have been described in patients who develop RCH following supratentorial craniotomy including altered level of consciousness, motor deficits, gait ataxia, and seizures [3]. Concern for edema and mass effect in the posterior fossa on the 4th ventricle can lead to sudden hydrocephalus and needs to be carefully monitored and urgently treated if it develops [1]. RCH may also present asymptomatically, and thus many patients with RCH proceed undiagnosed [3]. In these cases, follow-up imaging is essential to early detection. In a systematic review by Sturiale et al., symptomatic diagnosis of post-craniotomy RCH was made in 63% of reported cases and asymptomatic diagnosis in 37% of cases found incidentally on post-operative imaging [1]. Conversely, symptomatic diagnosis of post-spinal surgery RCH was made in 100% of cases, as no post-operative imaging was performed without complaint of neurologic symptoms [17]. Overall mortality in our reviewed cases were 16.7% and 14.3% for post-craniotomy and post-spinal surgery, respectively.

Interestingly, our review yielded only one asymptomatic case identified incidentally on follow-up imaging. It is possible minor intra-operative or post-operative CSF leaks present with milder symptoms due to less dramatic intracranial pressure changes. Konig et al. identified higher frequencies of RCH following resection of large supratentorial tumors and hypothesized that resection of larger masses confer greater reduction of intracranial pressure (ICP) and increased transmural venous pressure, increasing RCH risk [18]. However, it is curious then why RCH is not common after routine lumbar drain (LD) or EVD placement following cranial surgery or ventriculoperitoneal (VP) shunt placement for hydrocephalus. We hypothesize that rapid rates of large volumes of CSF leakage, and thus rate of ICP decline, combined with unique anatomical configurations of venous drainage, contribute to post-operative RCH and explain the lack of RCH reports following controlled CSF drainage.

The role of patient positioning in RCH pathogenesis is uncertain. In craniotomy cases with position reported, patients were commonly placed supine with the head rotated opposite the craniotomy [9]. Interestingly, in both of our cases, hemorrhage occurred in the cerebellar hemisphere contralateral to the direction of head rotation. This corroborates Papanastassiou, Adams, and Chir’s hypothesis that hemorrhage preferentially occurs contralateral to the craniotomy as the contralateral cerebellar hemisphere is directed posteriorly to abut and kink the transverse sinus following CSF leakage [2]. Cerebellar movement opposite the craniotomy site may also explain the higher occurrence of RCH in frontal, frontotemporal, or pterional craniotomies [4,6-10]. Additionally, “cerebellar sag” and compression of posterior venous sinuses may be further exacerbated by gravity with supine positioning. This underscores the rarity of our case presentation. Our cases are among the first to report RCH occurrence following suboccipital craniotomies in the prone position. With prone, suboccipital craniotomy, we postulate anterior cerebellar displacement is causing compression of anterior cerebellar veins, rather than compression of the transverse or sigmoid sinuses. Notably, the role of patient positioning in RCH following spinal surgeries has been insignificant [11,13,14]. Interestingly, all cases of RCH following spine surgery included in our review occurred following surgery at the lumbar level; this is consistent with prior literature showing the incidence of RCH is highest in surgeries involving lumbosacral segments compared to cervical and thoracic [17]. 

Our study highlights the importance of avoiding rapid excessive CSF loss during surgery, to reduce the risk of RCH peri-operative complications. The use of controlled CSF drainage techniques, including small controlled dural openings, provides favorable strategies to regulate peri-operative CSF drainage rates. If large losses of CSF are observed, patients should be carefully monitored and imaged post-operatively. Placing patients in reverse Trendelenburg can be performed if there is a concern for RCH after high-risk procedures, although no data supporting this method has been shown. For symptomatic patients with altered mental status and reduced consciousness, RCH secondary to hemorrhagic venous infarction should be suspected.

Our cases illustrate that RCH is not limited to supratentorial craniotomies. Our case following burr hole surgery represents a rare neurosurgical setting for post-operative RCH. To our knowledge, only two previous cases of RCH following burr hole evacuation of subdural hematomas have been described [19,20]. The proposed pathophysiology remains similar, massive and rapid evacuation of a space-occupying lesion resulting in a drastic decrease in ICP. These reports signify that post-operative RCH is not limited to craniotomies; careful monitoring remains essential in other procedures involving rapid and large volume evacuation or reduction in ICP. Thus, to avoid an overly rapid rate of CSF drainage following burr hole subdural hygroma evacuation, we recommend placing JP bulb to gravity suction rather than self-suction and keeping the patient supine post-operatively.

## Conclusions

Although RCH is largely considered a frontotemporal and frontal craniotomy complication, we report the first known case of RCH following suboccipital craniotomy. Additionally, we report one of the first cases of RCH following burr holes for subdural hygroma evacuation, demonstrating the risk of RCH even after less-invasive procedures. Although traditional descriptions of RCH etiology detail intra-operative over drainage of CSF, we hypothesize it is the rate of CSF drainage, rather than volume, that contributes to RCH pathogenesis. Controlled CSF drainage may prevent this occurrence from happening. Similarly, after burr holes for subdural hygromas, it may be wise to place JP drains to gravity rather than bulb suction.
